# Broad-scale informed consent: A survey of the CTSA landscape

**DOI:** 10.1017/cts.2019.397

**Published:** 2019-09-23

**Authors:** Redonna Chandler, Kathleen T. Brady, Rebecca N. Jerome, Milton Eder, Erin Rothwell, Kimberly A. Brownley, Paul A. Harris

**Affiliations:** 1 National Institute on Drug Abuse (NIDA), National Institutes of Health (NIH), Bethesda, MD, USA; 2 South Carolina Clinical and Translational Research Institute (SCTR), Medical University of South Carolina, Charleston, SC, USA; 3 Vanderbilt Institute for Clinical and Translational Research, Vanderbilt University Medical Center, Nashville, TN, USA; 4 Department of Family Medicine and Community Engagement to Advance Research and Community Health (CEARCH), University of Minnesota, Minneapolis, MN, USA; 5 College of Nursing, University of Utah, Salt Lake City, UT, USA; 6 Department of Psychiatry, University of North Carolina School of Medicine, Chapel Hill, NC, USA; 7 Department of Biomedical Informatics, Vanderbilt University School of Medicine, Nashville, TN, USA

**Keywords:** Informed consent, data sharing, biobanking, participant contact, electronic health records

## Abstract

**Introduction:**

Research opportunities associated with the proliferation of the electronic health record (EHR), big data initiatives, and innovative approaches to trial design can present challenges for obtaining and documenting informed consent. Broad-scale informed consent (a term used herein to describe institutional models, rather than the Common Rule’s strict regulatory definition for “broad consent”) may facilitate the use of existing data and samples and speed the pace of research by minimizing barriers to consent. We explored the use of broad-scale informed consent within the Clinical Translational Science Award (CTSA) Program Network.

**Methods:**

We surveyed CTSA Hubs concerning policies, practices, experiences, and needs within three domains of broad-scale informed consent: (1) participant recontact; (2) biospecimens; and (3) clinical data sharing.

**Results:**

Of 61 CTSA Hubs surveyed, 37 (61%) indicated ongoing work related to at least 1 domain of broad-scale informed consent; 18 Hubs (30%) reported work in all 3 domains. The EHR predominated as the implementation system across all three domains. Research and IT leadership and the Institutional Review Board were most commonly endorsed as institutional drivers, while systems/technical issues and impact on clinical workflow were the most commonly reported barriers.

**Conclusions:**

While survey results indicate considerable variability in the implementation of broad-scale informed consent across the CTSA consortium, it is clear that all CTSA Hubs are actively considering policy and process related to these concepts. Next steps cluster within three areas: training and workforce development, streamlined policies and templates, and implementation strategies that facilitate integration into clinical workflow.

## Introduction

Appropriate procedures for informed consent are widely recognized as an ethical mandate for research involving human participants.^1^ In a typical research context, informed consent is obtained for participation in a single, clearly delineated study and includes information related to disclosure, voluntariness, and comprehension of the study. Unless waived by an Institutional Review Board (IRB), study-specific consent is required for research projects involving interaction, intervention, and/or the collection of identifiable information about living research participants.

There is growing diversity in scientific investigation, beyond typical clinical trials, including large pragmatic trials involving comparison of effective interventions, e-health interventions, survey research, and the expansion of biospecimen banking.^2^ In addition, new opportunities associated with the proliferation of the electronic health record (EHR), creation of clinical data warehouses, and data science initiatives provide unprecedented opportunities to harness existing data to advance research to improve human health. These new opportunities can present new challenges for obtaining and documenting informed consent.

The overarching policy governing research with human subjects in the USA, known as the Common Rule, has recently been revised with provisions for broad consent as an alternative to traditional study-specific consent for human subjects research.^3^ This revision was finalized in 2017 and fully implemented in early 2019. Specifically, it is stated that: “Broad consent for the storage, maintenance, and secondary research use of identifiable private information or identifiable biospecimens (collected for either research studies other than the proposed research or nonresearch purposes) is permitted as an alternative to the informed consent requirements,” referring to sections describing traditional informed consent requirements outlined elsewhere in the Common Rule.^3^ The document contains multiple stipulations related to the use of broad consent including incorporating selected basic consent and additional elements such as describing what information/specimens will be used, if they will be shared, and the importance of having a system to track and honor individual consent decisions.

Beyond this specific regulatory definition, the current work conceptualizes broad consent more holistically as a process in which participants agree prospectively to have biological samples, genomic data, and/or health information retained for use in future research deemed appropriate by some oversight body, including IRB review. This article discusses policies and practices in place or in planning stages at institutions that are in the spirit of consent for future research activities, thus the phrase “broad-scale informed consent” is used to encompass a context broader than the more specific regulatory definition for “Broad Consent”. For example, “broad-scale informed consent” might include practices currently in use at institutions that are more conservative than current regulatory requirements, such as obtaining informed consent for use of de-identified data and specimens or contact for future research opportunities.

Use of such practices can facilitate the use of existing data and samples in research and speed the pace of research by minimizing practical barriers to getting research consent, particularly when the risk of harm to research participants is deemed low.^4^,^5^ Discussions concerning ethical considerations surrounding broad-scale informed consent have been active in biobanking for many years. Many agree that broad-scale informed consent is ethically acceptable as long as participant privacy is protected, and participants are provided sufficient information to make a reasonably informed decision.^2^ Surveys indicate support from potential research participants for this concept, though some subgroups, such as those from racial and ethnic minority groups and those with lower educational attainment, express significantly lower willingness to participate in such models.^6^^,^^7^ Thus, many questions about the acceptability and feasibility of broad-scale informed consent remain. Approaches to broad-scale informed consent under discussion include the use of an informed yes/no decision by potential participants, whereby individuals must explicitly state they agree to the use of biological samples, genomic data, and/or health information in future research. Other notable issues related to broad-scale informed consent include establishing an oversight process for subsequent research use, sharing of samples with other institutions, withdrawing individuals from further research, saving identifiable specimens, and subsequent reporting of new findings.^7^^,^^8^

Although many issues concerning broad-scale informed consent in biobanking remain, there is growing interest in expanding its use beyond biobanking to leverage opportunities associated with the transition to electronic processing of health care data.^2^^,^^9^ For example, during activities preparatory to research and with IRB oversight, individuals who have given consent for contact for future research activities and are potentially eligible for participation in a specific study could be identified through the EHR or other registry and invited to participate after consenting at the research project level. In this manner, broad-scale informed consent can facilitate the recruitment of participants into research and may be particularly useful for addressing significant gaps in recruiting minorities and women. Such gaps have long been recognized as a threat to clinical research and the advance of discovery to improve human health.^10^

Thus, there are a number of factors driving the use of broad-scale informed consent to facilitate research participation and improve the efficiency of biomedical research. However, it is critical to ensure ethical and pragmatic concerns are fully considered in implementation. With this in mind, we explored perceptions and practical use of broad-scale informed consent within the Clinical Translational Science Award (CTSA) Program Network.

## Methods

### Scope of Work

The National Institutes of Health began the CTSA Program in 2006 to “advance the assembly of institutional academic homes that can provide integrated intellectual and physical resources for the conduct of original clinical and translational science,” including the goal of providing a research environment that is “more nimble, conducive to, and responsive to the demands of modern translational and clinical research”.^11^

The Consent Working Group was established by the National Center for Advancing Translational Sciences (NCATS) and the CTSA Program Steering Committee in February 2017. At that time, there was widespread recognition that appropriately implementing broad-scale informed consent among individuals seeking care at CTSA Program Hubs and their affiliate health care settings was a critical step in catalyzing downstream research. Leadership from CTSA institutions further recognized that establishing a shared understanding of approaches to broad-scale informed consent within the CTSA consortium could contribute significantly to translational science by identifying best/successful practices for embedding research into clinical care and supporting the learning health care system.

A call for participants in this new working group was shared with the CTSA Steering Committee and the CTSA principal investigator (PI) and administrator email distribution lists. Approximately 20 volunteers representing 17 CTSA institutions, a representative from a community-based health care organization and representatives from NCATS formed the working group in spring 2017 to assess CTSA policies and practices related to broad-scale informed consent. The working group’s membership reflected the diversity of the CTSA Network, including representation of both public and private institutions, a variety of CTSA Program sizes and duration of Network participation, and professionals from a broad range of specialties and institutional positions.

In preparation for a CTSA-wide landscape survey, working group members from five CTSA organizations volunteered to present on activities at their institutions, specifically describing local planning and implementation activities, program goals, challenges, and successes. Relevant publications related to broad-scale informed consent were also collected and shared among the group.

Ultimately, working group discussions and presentations yielded three major domain areas of relevance: (1) consent to contact for future research (including necessary clinical data mining to establish potential study eligibility); (2) consent to use clinical data captured in EHR in a de-identified way for future research; and (3) consent to collect and use biospecimens for future research. Working group discussions also highlighted identification of institutional key stakeholders, institutional barriers to creation and implementation of broad consent policies, stakeholder communication strategies (e.g., patient groups), and post-implementation patient feedback.

The Consent Working Group sought to study current broad-scale informed consent practices across the CTSA Program with the goal of understanding current and planned practices, needs, and concerns, and with the goal of informing future efforts in the Program to facilitate multisite collaborative research and thus accelerate translation. To accomplish this goal and informed by the preceding activities of the group, the working group developed a survey to examine different approaches to broad-scale informed consent across the CTSA Program, determine if guidelines could be harmonized across the consortium, and evaluate the dissemination of broad-scale informed consent innovations currently underway at several Hubs.

### CTSA Network Survey

The working group developed an electronic survey to collect information related to broad-scale informed consent policies, practices, and experiences across the CTSA Network within the domains of: (1) participant recontact; (2) biospecimens; and (3) clinical data sharing. Survey design was iterative with input from the working group and pilot testing in three CTSA institutions; this feedback was used to further refine the questionnaire before deployment. The final survey instrument (see Supplemental Digital Appendix 1) was distributed to CTSA Hubs between August and October 2017; of note, this time period reflects a time during which the Common Rule changes had been finalized, but not yet implemented. The survey included 20 main questions and was estimated to take less than 30 minutes to complete. To reduce response burden and facilitate survey completion, the survey was designed to allow input from multiple individuals at a single institution. Study data were collected and managed using REDCap electronic data capture tools hosted at Vanderbilt University Medical Center.^12^ The IRB at Vanderbilt University determined that the survey met criteria for Exempt Review and approved the survey’s Request for Exemption on August 1, 2017 (IRB #171288).

Following input by the CTSA Steering Committee, the Consent Working Group project was discussed during a monthly CTSA PI call. The contact PI for each CTSA Network Hub received an email invitation asking them to complete or deputize one or more individuals within their organization to complete the broad-scale informed consent survey within 3 weeks. Institutions that did not respond within 2 weeks received a reminder and those institutions that had not responded by the survey deadline received a final reminder approximately 1 week later. Personal communication between Consent Working Group leaders and nonresponsive PIs was employed in a small number of instances to achieve 100% completion across all CTSA Program Hub institutions.

### Analysis

We summarized categorical and other quantitative data with descriptive statistics using R (R Foundation for Statistical Computing, Vienna, Austria) and Microsoft Excel (Microsoft Corporation, Redmond, Washington). We analyzed responses to open-ended questions using qualitative content analysis to explore themes as well as unique experiences.^13^

## Results

All 57 CTSA Hubs receiving funding in fiscal year 2017 and an additional 4 Hubs who had received funding during the previous fiscal year responded to the survey (see acknowledgment section for a list of institutions); all institutions provided substantive responses and were included in the analysis. CTSA Hubs were equal in self-reporting as publicly (*N* = 31) or privately funded (*N* = 30) institutions. The median number of individuals contributing to a Hub’s survey response was 1 (range 1–4, mean 1.7). A scan of institutional roles reported by respondents indicated that research and IRB/regulatory leadership largely predominated. Other respondent roles reflected contributions by IT leadership, research and regulatory staff, and a small number of medical ethics professionals.

The design of the survey was constructed to first determine whether an institution had an institutional policy for broad-scale informed consent in each of the three domains (participant contact, biospecimens, clinical data sharing). Allowable answers for each domain were “Yes,” “In Progress,” “Other” (with invitation to provide qualitative explanation), and “No.” Survey branching logic was designed to ask no further questions in a specific domain if the “do you have a policy?” question was answered “No,” but all non-“No” answers triggered a series of follow-up questions related to the specific domain. Altogether, 37 of 61 Hubs responded “Yes,” “In-Progress,” or “Other” to having a formal institutional policy for at least one of the three domains that constitute broad-scale informed consent for this study (Table 1).

**Table 1.** Broad-scale informed consent formal policy status

Response	Participant contact *N* (%)	Biospecimens *N* (%)	Clinical data sharing *N* (%)
Yes	17 (28)	13 (21)	16 (26)
In progress	13 (21)	10 (16)	8 (13)
Other*	1 (2)	2 (3)	2 (3)
No	30 (49)	36 (59)	35 (57)

* Qualitative assessment of “Other” responses generally suggested that the Hub’s guidance/practice derives not from direct engagement with broad-scale informed consent but from an appropriation of practices associated with study-specific research content (e.g., including within the informed consent document an option to grant researchers permission to contact the signee regarding future research).

Supplemental Digital Appendix 2 provides a breakdown of Hub broad-scale informed consent responses for ongoing (“Yes,” “In Progress,” “Other”) policy work across each of the three domains. Notably, 18 Hubs reported policy work in all 3 domains while 24 Hubs reported no policy work for any of the 3 domains.

In narrative sections below, we describe additional information collected from Hubs affirming policy work in the three broad-scale informed consent domain areas. Rather than organizing results in a domain-by-domain fashion (i.e., participant contact, biospecimens, clinical data sharing), we clustered reporting according to concepts that are shared across domains to provide a more holistic landscape view. Survey skip logic enabled Hub survey participants to answer only questions relevant to their local enterprise and current policy-related activities.

### Identified Versus De-identified

Supplemental Digital Content Appendix 3 provides information about the practice of storing and sharing de-identified versus identified data in biospecimen and clinical data sharing broad-scale informed consent domains (participant contact domain is excluded here as contact requires identified data storage/sharing). A majority of respondents indicated they had provisions for both identified and de-identified data in relation to clinical data sharing policies, while biospecimen-related policies were more evenly split between de-identified only approaches and sharing of both de-identified and identified data.

### Systems Utilization

Supplemental Digital Content Appendix 4 collates survey responses regarding the use of electronic systems to assist with the implementation of broad-scale informed consent policies. Respondents were encouraged to “check all that apply” in various categories shown as well as provide qualitative feedback when selecting the “Other” system category. Across all three domains of broad-scale informed consent, the EHR predominated as the most relevant implementation system, endorsed by 57% to 80% of Hubs, followed by patient portals, endorsed by 2058% of Hubs.

For respondents selecting “Other” for all three domains, in-person approaches were used to obtain broad-scale informed consent during clinical visits by either front desk or clinical staff. Hubs obtain and record either written or verbal consent to participant contact. “Other” comments further indicated support for consent during clinical encounters with brochure-based education materials handed to individuals or sent through an EHR patient portal. Hubs pursuing clinical data sharing reported using the EHR within the clinical encounter to flag patient records and track decisions as well as occasional use of clinical trial management systems for tracking patient preferences. For clinical data sharing, a few Hubs mentioned use of honest brokers as an option for centralizing records. Compliance strategies included potential IRB audits or frequent quality assurance checks; one Hub noted the difficulty in reliably excluding from recontact those patients who do not want to be asked about participating in research. A few Hubs indicated a lack of IT infrastructure impaired the capacity to record and retrieve patient broad-scale informed consent preferences.

### Alignment of Institutional Policy and Practice

Recognizing the difficulty of policy implementation in health care systems with competing priorities, we asked respondents with ongoing policy work to indicate on a slider bar from 0 (“Policy Not Yet Implemented”) to 100 (“Matches Policy Very Closely”) concordance between planned institutional policy and current institutional practice for each of the three broad-scale informed consent domains. Hubs reported high concordance overall with greatest concordance for participant contact (median = 86.5, range 51–100) and clinical data sharing (median = 89.5, range 0–100). There was slightly less policy and practice concordance for biospecimen management (median = 76.5, range = 1–100). Qualitative feedback indicated some Hubs have centralized biospecimen management that can facilitate ensuring concordance, while biospecimen collections at other Hubs remain with individual investigators and concordance is unknown. In addition, several Hubs commented on the absence of institutional-level policies regarding sharing clinical data or biospecimens, indicating current reliance in broad-scale informed consent documents on the language in the informed consent document for specific studies.

### Alignment with Changes to the Common Rule

Recognizing the difficulty of policy development and implementation of any regulatory requirement, we asked respondents with ongoing policy work to estimate current alignment between their institutional policy and finalized changes to the Common Rule that have yet to be implemented (Table 2). Overall responses suggest some alignment with Common Rule changes and clear areas for ongoing improvement. The survey did not explore whether discordance involves policy implementation or interpretation of finalized changes to the Common Rule.^3^ Results indicated a high degree of uncertainty regarding changes to the Common Rule and implications for broad-scale informed consent policies and procedures.

**Table 2.** Broad-scale informed consent practice compared with common rule changes

Response	Participant contact *N* (%)	Biospecimens *N* (%)	Clinical data sharing *N* (%)
Very closely	5 (16)	11 (44)	5 (19)
Somewhat closely	7 (23)	5 (20)	4 (15)
Not very closely	3 (10)	3 (12)	3 (12)
Not sure	11 (35)	5 (20)	9 (35)
No response	5 (16)	1 (4)	5 (19)

### Key Informants and Institutional Drivers

**Fig. 1.**
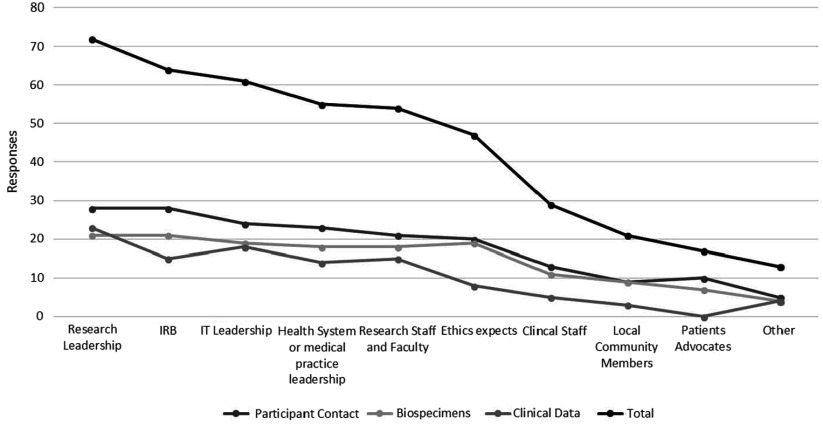
Key informants and institutional drivers of broad-scale informed consent policy and practice.

Hub respondents with ongoing policy work in each of the three domains were asked to provide information signifying key informants and drivers of broad-scale informed consent policies and practice at their institution. Figure 1 depicts these data in line graph formation to highlight the relative importance of various informants/drivers across all domains. “Other” responses identified compliance and legal personnel as primary drivers.

### Barriers and Facilitators to the Implementation of Broad-Scale Informed Consent

**Fig. 2.**
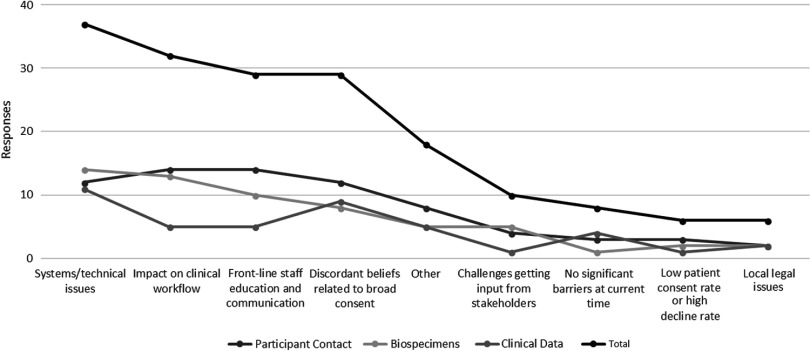
Key implementation barriers for broad-scale informed consent policies and practices.

Hub respondents with ongoing policy work in each of the three domains were also asked to identify key barriers to full actualization of institutional policies. Figure 2 depicts these data in line graph formation to highlight the relative weight of all barriers across all domains.

Qualitative data regarding facilitators and barriers to institutional broad-scale informed consent activities reveal several common themes. Facilitating factors included engagement and support of stakeholders (leadership, staff, patients, community), strong IT planning and resources, effective integration into clinical workflow, educational materials for patients, and frequent training for frontline staff. Conversely, key barriers include lack of stakeholder engagement, a lack of clarity regarding who should lead planning and operations for broad-scale informed consent implementation, and tension between the missions of clinical care and research. Other institutional barriers include process issues (e.g., variability in frontline practices; problems linking multiple IT systems), establishing broad-scale informed consent as a priority with IT colleagues (particularly during EHR system transitions), and limited resources to reach patients across the institution.

Respondents also shared lessons learned, across all three broad-scale informed consent domains. Shared key lessons included having knowledgeable staff to assist with consent during clinical encounters; education of potential participants and staff; and minimizing interference with clinical care workflows. Several Hubs also noted an institutional challenge due to multiple biorepositories within the Hub, tracking patient preferences, uncertainty about when consent is needed, and IT challenges. Qualitative responses from the 24 institutions reporting no current broad-scale informed consent activities revealed similar barriers and challenges.

### Researcher Access to Information and Materials

Recognizing that policy and practice around broad-scale informed consent are only useful if local researchers have access to resulting data, Hub respondents active in any of the three domains used a slider bar to indicate their institution’s progress in providing researchers access to data (100-point scale with 0 representing “not very clear or well-understood path,” 50 representing “…somewhat clear and well-understood path,” and 100 representing “… very clear and well-understood path”). Quantitative assessment yielded a median response of 51.5 (min = 0, max = 100), suggesting there is room for improving researcher understanding and access to data and specimens.

### Educational Strategies for Patients and Staff

Hubs with policy work in any of the three broad-scale informed consent domains were asked to describe local approaches to patient education around broad-scale informed consent. Common approaches include written materials (consent form, brochures/FAQs, poster), training of frontline staff and having them available to answer questions, and a dedicated phone line for questions. Fewer Hubs indicate using web-based materials (e.g., in patient portal), videos, in-clinic education modules, focus groups, or community advisory boards. Hubs emphasized the need for improving education for clinical staff because they interact most with patients and can obtain consent within the clinical encounter (e.g., in-person and/or access to web-based education). Qualitative responses stressed the need for both initial and refresher frontline staff training for continuity of consenting practices and documentation, as well as for reinforcing the importance of this activity as part of the research component of the institution’s mission.

### Special Populations and Considerations

Hubs with policy work in any of the three domains were asked to describe broad-scale informed consent efforts related to special populations. Several institutions have provisions for including children through parent/guardian consent; one institution reported excluding minors and another reported using specialized scripts to consent patients with diminished capacity with the assistance of their legally authorized representative. Some Hubs make broad-scale informed consent materials available in other languages to facilitate participation by non-English-speaking patients. Some Hubs also include language in the consent about creating living cell lines and excluding samples from research involving human cloning.

### Needs Assessment/Potential CTSA Network Initiatives

**Fig. 3.**
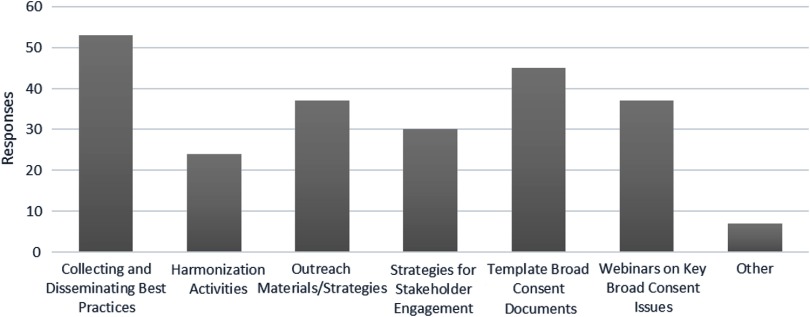
Recommended areas for collective action by the Clinical Translational Science Award consortium.

All survey respondents (with or without current broad-scale informed consent policy work in any domain) were asked: “How might the CTSA Network help facilitate broad-scale informed consent policies and/or implementation?”. The survey options and responses are listed in Fig. 3; Hubs were instructed to select all that apply. Many qualitative comments reinforced the options provided and desire for network-wide implementation.

## Discussion

This mixed quantitative/qualitative assessment provides a single, time-sensitive snapshot of local policy formulation and implementation of broad-scale informed consent in three domain areas (participant contact, biospecimens, clinical data sharing).

This is one of the first studies to survey institutions across the USA about broad-scale consent policies and practices. Most research has focused on participant attitudes toward different consent approaches and policies within genomic research and biobanks.^6^^,^^14^ Key findings of the current CTSA-wide survey indicate institutions are heterogeneous in their approach to formulating and implementing broad-scale informed consent policy. Progress (or intent to progress) on implementing broad-scale informed consent often varied by domain within a single institution, though implementation was slightly more common for the participant contact domain than biospecimen or clinical data use domains. Many institutions have regulatory-informed policies for handling both identifiable and de-identified biospecimens and clinical data, although a notable number of institutions use only de-identified policies for the future use of biospecimens.

The importance of leadership engagement and alignment across multiple areas (research, IRB, IT, health system, medical practice) was recognized as a key contributing factor for successful formulation and implementation of broad-scale informed consent policies. Ethics engagement and support was noted as a major factor in formulating biospecimen broad-scale informed consent policy.

Hubs reported success at obtaining broad-scale informed consent during clinical encounters but identified education/training of clinical staff as a weakness. Clear communication to staff about “why” broad-scale informed consent is important in addition to “how” to obtain broad-scale informed consent is needed given the potential for lower prioritization of research among those focused on clinical care. An institutional commitment to continually invest in training the consent workforce should be factored into policy and procedures. The current survey did not further query respondents for detailed information regarding staff education processes currently in place, or those discarded due to lack of utility; future work could delve further into these issues and explore opportunities for sharing educational approaches and lessons learned related to implementation and maintenance of broad-scale informed consent.

Qualitative survey responses indicated key support strategies, including capacity and confidence for staff and patients to reliably record/track participant decisions over time, clear understanding of practical use of honest brokers plus auditing processes to buttress confidentiality protections, and use of educational materials (e.g., brochures, videos) shared directly or via the patient portal. The literature on needs of research participants echoes some of these challenges, indicating difficulty in understanding the potential nature and scope of future unspecified research.^7^

Lack of system/technical implementation workflow was commonly cited as a barrier to broad-scale informed consent implementation. Based on survey results, systems support for EHR clinical workflow, patient portals, and/or clinical trial management systems are used to support consent tracking and patient preferences. Hubs indicated barriers to tracking participant decisions, effective stakeholder engagement, and education of both investigators and participants.

Survey responses indicated considerable uncertainty about implementation and institutional alignment with changes to the Common Rule, especially in areas of clinical data sharing and participant contact. Given the need for ongoing work to align institutional policies and implementation procedures with the Common Rule prior to implementation, this is an important area for training and education.

### Potential Limitations

We note several limitations that constrain the interpretation of survey results in this landscape analysis. In line with uncertainty preceding the implementation of the revised Common Rule, the survey results suggest potential lack of understanding among survey participants regarding the broad consent Common Rule policy that may have influenced responses to other questions. Our survey development focused on clarity and content validity, and did not assess response reliability (e.g., test/retest method). Further, while multiple individuals were able to contribute to an institution’s survey response in a collaborative fashion, our approach did not capture roles or compare relative contributions of individual respondents at the same institution.

Our data also indicated some incommensurability of qualitative and quantitative information, seemingly due to grandfathered programs (e.g., institutional biorepository versus legacy lab biorepository programs). For example, qualitative responses suggested that a few institutions were describing departmental-level and/or large researcher-lab policies rather than broad-scale informed consent policies related to participant contact, biospecimens, and clinical data sharing. For example, while some Hubs referred to a single policy implemented across the entire institution, others included consenting policies ranging from multiple institutional repositories to study-level consents for future contact and future research samples. Qualitative data were also relatively sparse, precluding any subgroup analysis such as exploring themes across the various nuances of institutional roadblocks and concerns. The survey also did not explore return on investment for institutions adopting broad-scale informed consent policies.

We also note that this survey sample focused on CTSA Hub institutions and did not include other institutions beyond the CTSA Network. However, many of the common issues described in the survey results, including barriers and challenges, importance of institutional leadership, degree of technological infrastructure, are not unique to the CTSA Network and are likely to be generalizable beyond its bounds to other institutions interested in broad-scale informed consent activities to support research.

## Conclusions

The use of broad-scale informed consent is an emerging approach intended to facilitate the efficient conduct of biomedical research in an ethical way that ensures respect for persons. Data collected via the survey described above provide a foundation for developing short- and long-term activities to be undertaken by CTSA Program consortium to move broad-scale informed consent forward.

Potential areas for activity inspired by the results of this survey fall within three broad areas: training and workforce development, streamlined policies and templates related to broad-scale informed consent, and implementation strategies that facilitate integrating broad-scale informed consent into clinical workflow. Following a review of the survey results, Working Group members consider developing training and educational materials and developing streamlined policies and templates related to broad-scale informed consent as important and reasonable next steps. Clearly, education and training are needed to ensure changes to Common Rule are reflected in local broad-scale informed consent policies and procedures. In addition, respondents identified educational materials for clinical staff and patients regarding broad-scale informed consent as a critical component to addressing barriers. Developing streamlined policies and templates for use across the CTSA Program consortium, and at other institutions pursuing these activities, could also facilitate the uptake of broad-scale informed consent. A near term step toward this process would be to collect policies and consent documents currently in use at Hubs for each of the three domains. Some, but not all, Hubs provided this information as part of the survey. Following the collection of this information, comparisons could reveal commonalities and differences, as well as set the stage for developing acceptable streamlined language for potential implementation into policies and broad-scale informed consent documents. Finally, members of the Working Group consider work on implementation strategies and integration of broad-scale informed consent into clinical workflow as premature and require intimate knowledge of the unique contextual variables within each CTSA Program Hub and affiliated medical settings.

## Participating Sites

We acknowledge with appreciation all CTSA Hub Sites contributing to the survey’s 100% response rate, including Albert Einstein College of Medicine, Boston University Medical Campus, Case Western Reserve University, Children’s National Medical Center, Columbia University, Dartmouth University, Duke University, Emory University, Georgetown University, Harvard Medical School, Icahn School of Medicine at Mount Sinai, Indiana University-Purdue University at Indianapolis, Johns Hopkins University, Mayo Clinic, Medical College of Wisconsin, Medical University of South Carolina, New York University School of Medicine, Northwestern University, Oregon Health Sciences University, Pennsylvania State University, Rockefeller University, Scripps University, Stanford University, SUNY Buffalo, The Ohio State University, Tufts University, University of Alabama Birmingham, University of California Davis, University of California Irvine, University of California Los Angeles, University of California San Diego, University of California San Francisco, University of Chicago, University of Cincinnati, University of Colorado Denver, University of Florida, University of Illinois at Chicago, University of Kansas Medical Center, University of Kentucky, University of Massachusetts Med School Worcester, University of Miami, University of Michigan, University of Minnesota, University of New Mexico, University of North Carolina Chapel Hill, University of Pennsylvania, University of Pittsburgh, University of Rochester, University of Southern California, University of Texas Health Science Center Houston, University of Texas Health Science Center San Antonio, University of Texas Med Br Galveston, University of Texas Southwestern, University of Utah, University of Washington, University of Wisconsin-Madison, Vanderbilt University, Wake Forrest, Washington University, Weill Cornell Medical Center, Yale University.

## Supplementary material

For supplementary material accompanying this paper visit https://doi.org/10.1017/cts.2019.397. click here to view supplementary material
