# Supplementary material for: Broad-scale informed consent: A survey of the CTSA landscape
Source: J Clin Transl Sci. 2019 Sep 23;3(5):253–60. doi: 10.1017/cts.2019.397 (PMC6813518; doi:10.1017/cts.2019.397)
Supplement: Supplementary file 1 [file S2059866119003972sup.zip › S2059866119003972sup001.pdf]

Supplemental Digital Content Appendix 1:
CTSA Broad-Scale Informed Consent
Survey

CTSA Broad Consent Survey

Please complete the survey below.

Thank you!

This survey, being sent to all CTSA Hubs, is intended to capture experiences with institutional policies and practices related to "broad consent" in key areas, and to assess the current landscape in the CTSA Network and identify opportunities for Network activities to support Hubs and their partners.

Please answer the questions below thinking about policies and practices at your institution. We have also included a question at the end of the survey in which you will have the option to note any relevant broad consent activities at your Hub's partner institutions.

For the purposes of this survey, we are using the inclusive operational definition for broad consent developed by Grady et al.: "We define "broad consent" as consent for an unspecified range of future research subject to a few content and/or process restrictions." (Grady et al., Am J Bioeth. 2015; 15(9): 34-42.)

Note: due to the survey's use of branching logic, questions may not be contiguously numbered.

You may also deputize colleagues at your institution to contribute information to this survey. To avoid overwriting data, only one person at a given time can actively edit your institution's survey. After 60 minutes the link will reactivate allowing others to edit the survey.

IMPORTANT: Please use the "submit" button at the bottom of the survey to save your changes before closing this window.

0. Institutional delegate(s)

Is there anyone else at your institution that should receive an invitation to fill out this survey?
◯ Yes
◯ No

Please enter their email address
____

Is there anyone else at your institution that should receive an invitation to fill out this survey?
◯ Yes
◯ No

Please enter their email address
____

Is there anyone else at your institution that should receive an invitation to fill out this survey?
◯ Yes
◯ No

Please enter their email address
____

Is there anyone else at your institution that should receive an invitation to fill out this survey?
◯ Yes
◯ No

Please enter their email address
____

Is there anyone else at your institution that should receive an invitation to fill out this survey?
◯ Yes
◯ No

Please enter their email address

____

1.Does your institution have a formal policy describing broad consent in these areas?

1a. Participant contact (throughout this survey, this refers to permission to contact patient as a potential research participant)

- ◯ Yes
- ◯ No
- ◯ Not yet (in progress)
- ◯ Other

Please describe (other approach to participant contact broad consent):

____

Optional: If you'd like to share your institution's broad consent policy on participant contact, please upload here.

All policies submitted will be comparatively explored for common wordings and practices.

1b. Biospecimens (throughout this survey, this refers to biospecimens collected during clinical care)

- ◯ Yes
- ◯ No
- ◯ Not yet (in progress)
- ◯ Other

Please describe (other approach to biospecimen broad consent):

____

What type(s) of information sharing does your institution's biospecimen broad consent allow?

- ◯ De-identified/non-identified
- ◯ Identifiable
- ◯ Both identifiable or de-identified
- ◯ Other

Please describe (other biospecimen information sharing):

____

Optional: If you'd like to share your institution's broad consent policy for biospecimens, please upload here.

All policies submitted will be comparatively explored for common wordings and practices.

1c. Clinical data sharing

- ◯ Yes
- ◯ No
- ◯ Not yet (in progress)
- ◯ Other

Please describe (other approach to clinical data sharing):

____

What type(s) of information sharing does your institution's clinical data sharing broad consent allow?

◯ De-identified/non-identified
◯ Identifiable
◯ Both identifiable or de-identified
◯ Other

Please describe (other type of clinical data sharing):

____

Optional: If you'd like to share your institution's broad consent policy for clinical data sharing, please upload here.

All policies submitted will be comparatively explored for common wordings and practices.

2. How closely does your institution's current implementation of broad consent match relevant institutional policy in each of these three areas? (For example, if your institution has worked through most key challenges related to obtaining broad consent and implementation now tightly matches the policy, you may use the slider to select a point close to the "very closely" end of the spectrum. If some key challenges remain at your institution, you may select a point closer to the middle of the slider bar.)

2a. Participant contact

Not yet implemented | Implementation somewhat matches policy | Implementation matches policy very closely

(Place a mark on the scale above)

2b. Biospecimens

Not yet implemented | Implementation somewhat matches policy | Implementation matches policy very closely

(Place a mark on the scale above)

2c. Clinical data sharing

Not yet implemented | Implementation somewhat matches policy | Implementation matches policy very closely

(Place a mark on the scale above)

3. How is your institution implementing broad consent (or planning to implement, if currently in progress)? Please select all that apply.

3a. Participant contact

☐ Clinical trial management system
☐ Electronic health record
☐ Billing system
☐ Patient portal
☐ Other

Please describe:

____

Please describe your institution's process (including planned process, if not yet implemented) for broad consent related to participant contact, including what staff are involved, when and where broad consent is obtained, and other features of this process.	__________________________________
3b. Biospecimens	☐ Clinical trial management system<br>☐ Electronic health record<br>☐ Billing system<br>☐ Patient portal<br>☐ Other
Please describe:	__________________________________
Please describe your institution's process (including planned process, if not yet implemented) for broad consent related to biospecimens, including what staff are involved, when and where broad consent is obtained, and other features of this process.	__________________________________
3c. Clinical data sharing	☐ Clinical trial management system<br>☐ Electronic health record<br>☐ Billing system<br>☐ Patient portal<br>☐ Other
Please describe:	__________________________________
Please describe your institution's process for broad consent related to clinical data sharing, including what staff are involved, when and where broad consent is obtained, and other features of this process.	__________________________________

4. What is your current (or planned) approach to broad consent in each area?

4a. Participant contact	◯ Opt-in<br>◯ Opt-out<br>◯ Mixed approach<br>◯ Other
Please describe:	__________________________________
4b. Biospecimens	◯ Opt-in<br>◯ Opt-out<br>◯ Mixed approach<br>◯ Other
Please describe:	__________________________________

4c. Clinical data sharing

◯ Opt-in
◯ Opt-out
◯ Mixed approach
◯ Other

Please describe:

____

5. Who has been involved as key informants and/or drivers of broad consent policies and practices at your institution (for each area)? Please select all that apply.

5a. Participant contact

☐ Research leadership
☐ Research staff and faculty
☐ Local community members
☐ Clinical staff
☐ Patient advocates
☐ IRB
☐ Ethics experts
☐ Health system or medical practice leadership
☐ IT leadership
☐ Other

Please describe:

____

5b. Biospecimens

☐ Research leadership
☐ Research staff and faculty
☐ Local community members
☐ Clinical staff
☐ Patient advocates
☐ IRB
☐ Ethics experts
☐ Health system or medical practice leadership
☐ IT leadership
☐ Other

Please describe:

____

5c. Clinical data sharing

☐ Research leadership
☐ Research staff and faculty
☐ Local community members
☐ Clinical staff
☐ Patient advocates
☐ IRB
☐ Ethics experts
☐ Health system or medical practice leadership
☐ IT leadership
☐ Other

Please describe:

____

6. What barriers are preventing full actualization of your institution's policies related to broad consent (for each area)? Please select all that apply.

6a. Participant contact

- ☐ Challenges getting input from relevant stakeholders
- ☐ Systems/technical issues
- ☐ Front-line staff education and communication
- ☐ Low patient consent rate or high decline rate
- ☐ Discordant beliefs related to broad consent among institutional stakeholders
- ☐ Local legal issues
- ☐ Impact on clinical workflow
- ☐ Other
- ☐ No significant barriers at current time (i.e., broad consent is fully actualized and barriers have been overcome)

Please describe: ______________________________

6b. Biospecimens

- ☐ Challenges getting input from relevant stakeholders
- ☐ Systems/technical issues
- ☐ Front-line staff education and communication
- ☐ Low patient consent rate or high decline rate
- ☐ Discordant beliefs related to broad consent among institutional stakeholders
- ☐ Local legal issues
- ☐ Impact on clinical workflow
- ☐ Other
- ☐ No significant barriers at current time (i.e., broad consent is fully actualized and barriers have been overcome)

Please describe: ______________________________

6c. Clinical data sharing

- ☐ Challenges getting input from relevant stakeholders
- ☐ Systems/technical issues
- ☐ Front-line staff education and communication
- ☐ Low patient consent rate or high decline rate
- ☐ Discordant beliefs related to broad consent among institutional stakeholders
- ☐ Local legal issues
- ☐ Impact on clinical workflow
- ☐ Other
- ☐ No significant barriers at current time (i.e., broad consent is fully actualized and barriers have been overcome)

Please describe: ______________________________

Following up on your answers to 6 a, b, and c: Please describe the nature of each of these challenges at your institution. Your insights may lead to additional opportunities for the CTSA Network to facilitate broad consent efforts in the future. ______________________________

7. Please describe any other characteristics about your institution that affect its approach to broad consent (e.g., governance issues related to multiple separate entities within a health system, other infrastructure challenges).

8. Please share any descriptive statistics about broad consent implementation at your institution, such as number of patients at your institution that have provided broad consent, the proportion of the institution's patient population that has provided broad consent, the proportion of patients who have refused to provide consent or opted out, etc.

9. Please share any observations about barriers and/or facilitators to the on-the-ground process for successfully obtaining broad consent from patients at your institution.

10. Please describe any lessons learned at your institution, positive and/or negative, related to broad consent for:

10a. Participant contact: __________________________________

10b. Biospecimens: __________________________________

10c. Clinical data sharing: __________________________________

11. If your broad consent policies address any special populations, permissions, or uses (e.g., stem cells, other potentially sensitive use cases), please describe.

12. Please generally characterize your institution's process for researcher access to data covered by broad consent.

Not very clear or well-understood path	Somewhat clear and well-understood path	Very clear and well understood path

(Place a mark on the scale above)

13. How closely does your institution's broad consent align with the planned Common Rule changes?

13a. Participant contact

- ◯ not very closely
- ◯ somewhat closely
- ◯ very closely
- ◯ not sure

13b. Biospecimens

- ◯ not very closely
- ◯ somewhat closely
- ◯ very closely
- ◯ not sure

13c. Clinical data sharing

- ◯ not very closely
- ◯ somewhat closely
- ◯ very closely
- ◯ not sure

14. How does your institution ensure that patients who opt out or decline broad consent are not contacted and/or their data is not employed in research protocols that utilize your broadly-consented population?

15. What educational strategies has your institution implemented to inform potential research participants about broad consent? Please include who answers potential participant questions about broad consent (e.g., nurses, registration staff, etc.).

16. How might the CTSA Network help facilitate broad consent policies and/or implementation? Please select all that apply.

- ☐ Collecting and disseminating recipe/best practices
- ☐ Harmonization activities
- ☐ Outreach materials/strategies for various stakeholder groups (e.g., patients, frontline staff)
- ☐ Strategies for stakeholder engagement (e.g. community members, researchers, staff)
- ☐ Template broad consent documents
- ☐ Webinars discussing key issues relevant to broad consent (e.g. common rule changes, models, engagement activities)
- ☐ Other

Please describe:

____

17. Please share who was involved in completing this survey, including corresponding role(s) at the institution:

18. Your institution's name:

____

19. We would also like to better understand local context factors that define the value proposition for broad consent at your institution. Please share available data describing your institution related to the following:

19a. Your institution's size, related to NIH funding:

- ◯ Small
- ◯ Medium
- ◯ Large

19b. Please indicate your institution type:

◯ Public institution
◯ Privately funded institution

20. In this round of input, we are focusing on the CTSA Hubs, but will identify opportunities for further follow-up in the future. If you are aware of interesting work related to broad consent at any of your Hub's partner institutions, please let us know here:

Please use the "submit" button below to save your edits. Your link to the survey will still remain active, should you need to return later to add or edit your responses.

To help us understand when the data is ready for analysis, please check the box below when this survey is complete for your institution.

☐ survey is complete
