# Supplementary material for: Broad-scale informed consent: A survey of the CTSA landscape
Source: J Clin Transl Sci. 2019 Sep 23;3(5):253–60. doi: 10.1017/cts.2019.397 (PMC6813518; doi:10.1017/cts.2019.397)
Supplement: Supplementary file 1 [file S2059866119003972sup.zip › S2059866119003972sup002.pptx]

## Slide 1
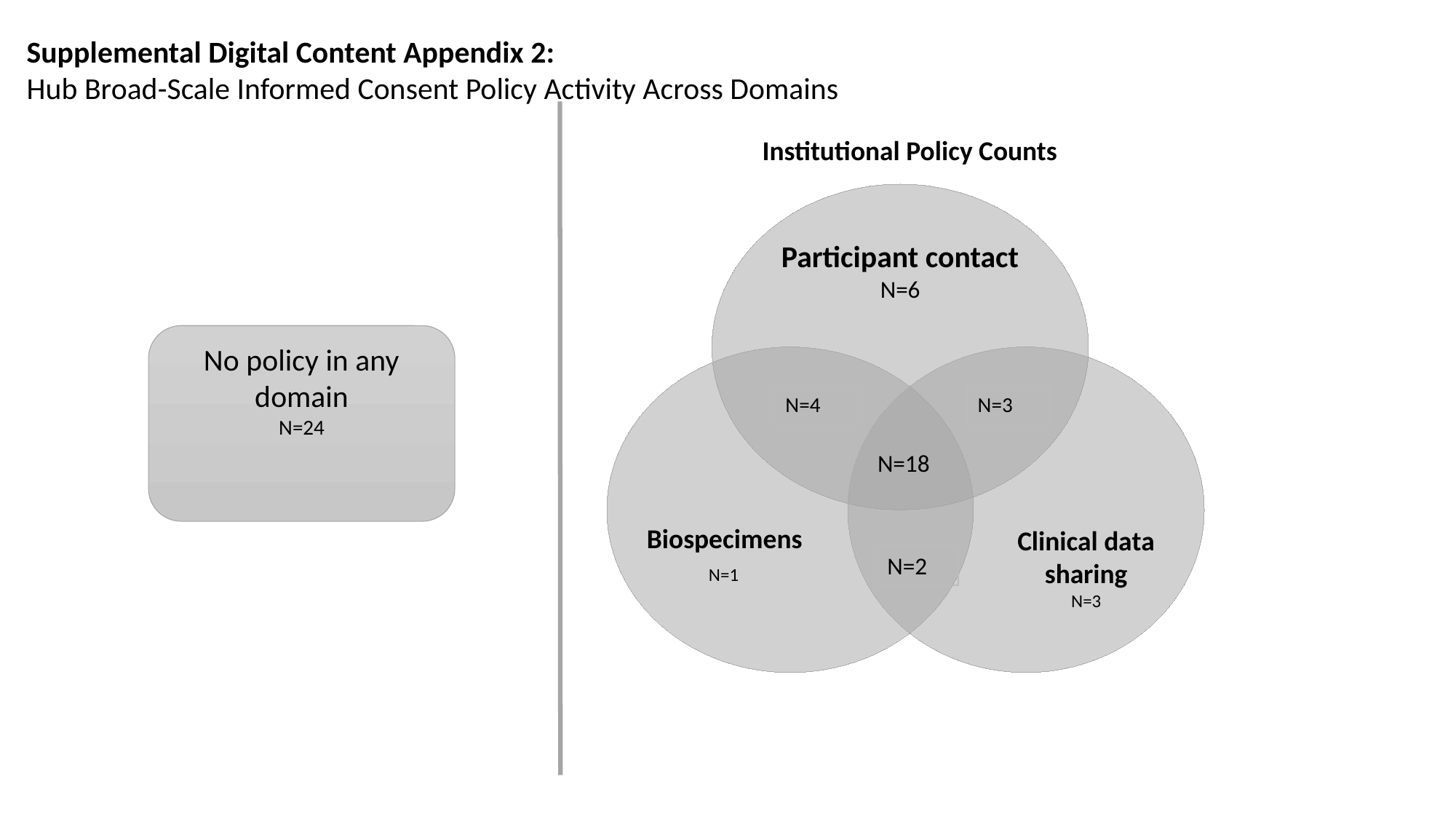

Supplemental Digital Content Appendix 2:
Hub Broad-Scale Informed Consent Policy Activity Across Domains
| | | | | | | | | | | | | |
| --- | --- | --- | --- | --- | --- | --- | --- | --- | --- | --- | --- | --- |
| | | | | | | | | | | | | |
| | | | | | | | Institutional Policy Counts | | | | | |
| | | | | | | | | | | | | |
| | | | | | | | | | | | | |
| | | | | | | | | | | | | |
| | | | | | | | | | | | | |
| | | | | | | | | | | | | |
| | | | | | | | | | | | | |
| | | | | | | | | | | | | |
| | | | | | | | | | | | | |
| | | | | | | | | | | | | |
| | | | | | | | | | | | | |
| | | | | | | | | | | | | |
| | | | | | | | | | | | | |
| | | | | | | | | | | | | |
| | | | | | | | | | | | | |
| | | | | | | | | | | | | |
| | | | | | | | | | | | | |
| | | | | | | | | | | | | |
| | | | | | | | | | | | | |
| | | | | | | | | | | | | |
| | | | | | | | | | | | | |
| | | | | | | | | | | | | |
| | | | | | | | | | | | | |
| | | | | | | | | | | | | |
| | | | | | | | | | | | | |
Participant contact
N=6
No policy in any domain
N=24
N=4
N=3
N=18
Biospecimens
 N=1
Clinical data sharing
N=3
N=2
