# Supplementary material for: Broad-scale informed consent: A survey of the CTSA landscape
Source: J Clin Transl Sci. 2019 Sep 23;3(5):253–60. doi: 10.1017/cts.2019.397 (PMC6813518; doi:10.1017/cts.2019.397)
Supplement: Supplementary file 1 [file S2059866119003972sup.zip › S2059866119003972sup003.docx]

**Supplemental Digital Content Appendix 3:** Identification/De-identification in Specimen and Data Sharing

| **Response** | **Biospecimens**  **(25 Hubs)**  **N (%)** | **Clinical Data Sharing**  **(26 Hubs)**  **N (%)** |
| --- | --- | --- |
| De-identified storage/sharing only | 10 (40%) | 6 (23%) |
| Identified and de-identified storage/sharing allowed | 12 (48%) | 16 (62%) |
| ‘Other’ or no response | 3 (12%) | 3 (12%) |
