# Supplementary material for: Broad-scale informed consent: A survey of the CTSA landscape
Source: J Clin Transl Sci. 2019 Sep 23;3(5):253–60. doi: 10.1017/cts.2019.397 (PMC6813518; doi:10.1017/cts.2019.397)
Supplement: Supplementary file 1 [file S2059866119003972sup.zip › S2059866119003972sup004.docx]

**Supplemental Digital Content Appendix 4:**  Broad-Scale Informed Consent Implementation Systems

| **Domain** | **Electronic Health Record**  **N (%)** | **Patient Portal**  **N (%)** | **Clinical Trial Management System (CTMS)**  **N (%)** | **Billing System**  **N (%)** | **Other**  **N (%)** |
| --- | --- | --- | --- | --- | --- |
| Participant Contact (N=31) | 22 (71%) | 18 (58%) | 7 (23%) | 0 | 9 (29%) |
| Biospecimen (N=25) | 13 (57%) | 8 (35%) | 5 (22%) | 0 | 10 (44%) |
| Clinical Data (N=26) | 20 (80%) | 5 (20%) | 6 (24%) | 0 | 7 (28%) |
